# Evaluation of two treatment concepts of four implants supporting fixed prosthesis in an atrophic maxilla: finite element analysis

**DOI:** 10.1186/s12903-023-03706-4

**Published:** 2023-12-08

**Authors:** Anlin Li, Zhixiang Mu, BaiRui Zeng, Tianxi Shen, Rongdang Hu, Huining Wang, Hui Deng

**Affiliations:** School and Hospital of Stomatology, Wenzhou Medical University, Wenzhou, 325027 PR China

**Keywords:** Dental implants, Implant supported dental prosthesis, Finite element analysis, Short implant, Title implant

## Abstract

**Background:**

Currently, oblique placement of long implants or the use of short implants to circumvent the maxillary sinus area and provide support for fixed prostheses are viable alternatives. The purpose of this study was to compare these two treatment concepts and ascertain which one exhibits superior biomechanical characteristics.

**Methods:**

Two different treatment concept models were constructed. The first one, LT4I, consisting of two mesial vertical implants positioned in lateral incisor regions and two distal tilted implants (45°) situated in second premolar regions of the maxilla. The second model, VS4I, includes two mesial vertical implants in lateral incisor regions and two vertically positioned short implants in second premolar regions. Numerical simulations were conducted under three loading types: firstly, oblique forces upon the molars; secondly, vertical forces upon the molars; thirdly, oblique forces upon the incisors. The maximum principal stress (σ_max_) and minimum principal stress (σ_min_) of the bone, as well as von Mises stress of the implants, were calcuated.

**Results:**

Under oblique loading on the molar, higher stress values in the bone were observed in LT4I group. Under vertical loading on molar, higher stress values in the bone were also observed in LT4I group. Furthermore, little difference was found between the two groups under oblique loading on the incisor.

**Conclusion:**

Both treatment concepts can be applicable for edentulous individuals with moderate atrophic maxilla. Compared to tilted implants, short implants can transmit less occlusal force to the supporting tissues.

## Introduction

The fixed prosthesis of atrophic jaws with dental implants has been considered a clinical challenge [1], as it involves complex three-dimensional (3D) anatomic structure such as the maxillary sinus and nasal cavity, making surgical procedures more difficult [2]. Moreover, the process of reabsorption in the maxilla results in both vertical and horizontal loss of alveolar bone, and tooth loss can further lead to sinus pneumatization, adding to the complexity of the surgery [2, 3]. Additionally, the observation of low bone quality and volume in moderate atrophic maxilla is quite common [4]. These potential complications significantly restrict the width, number, length, and positioning of the implants to be used, ultimately affecting the final treatment outcome [5]. To make implantation in posterior regions possible, maxillary sinus elevation and bone grafting are commonly performed at the implant placement site to restore bone height [3, 6]. However, this approach carries a high risk of surgical complications, such as infection and perforation of the maxillary sinus mucosa, as well as increased costs and prolonged treatment duration [1, 7].

P Maló et al. suggested that employing four implants (two in the anterior region, and two tilted ones in the posterior region of the alveolar bone, named all-on-4) could serve as an alternative to bone grafting procedures [8]. Clinical studies have shown promising results for the all-on-4 treatment concepts [9, 10]. Furthermore, studies have demonstrated that the use of short implants may also serve as a viable alternative to bone graft surgeries, with favorable clinical outcomes [6, 11]. Both treatment concepts have high implant survival rate [10, 12]. Nevertheless, we remain unaware of the superior treatment approach, and it behooves us to employ finite element analysis (FEA) to assess the two above treatment.

Numerous scholars have conducted FEA of various treatment options for fixed prosthesis. Initially, Chiara M.Bellini et al. used simplistic models to compare the effects of all-on-4 and all-on-5 (five vertical implants) on the mandible, revealing no significant disparity in stress values [13]. A.S. Bonnet et al. demonstrated that the location of food has an effect on stress concentration in bone for the all-on-4 concept, especially on bone-implant interface [14]. Afterwards, several scholars explored the impact of implant angles and cantilever length in all-on-4 treatment. They found that the use of tilted implants can cause increased stress on the cortical bone around the implants. And the use of tilted implants combined with a short cantilever in the all-on-4 concept reduces the stress around the implant on cortical bone [15, 16]. While previous researches have predominantly focused on all-on-4 concept, scant attention has been paid to the application of short implants in the atrophic maxilla. In recent biomechanical studies, Erika O. Almeida group and Cláudia Lopes Brilhante Bhering group explored the utilization of short implants in edentulous maxilla [1, 5]. However, it is worth noting that the short implants used in the posterior maxilla in these studies followed the all-on-6 treatment concept rather than the all-on-4 treatment concept. Therefore, when bone height is limited in the posterior region, it remains uncertain whether the placement of short implants surpasses that of tilted implants. Furthermore, their studies could be enhanced by considering the anisotropic nature of bone properties [14]. And the occlusal force referred in previous studies only set for molars but without incisors [5, 17].

This study, via different bite loading settings and appropriate anisotropic parameters of bone, compared the biomechanical property of these two treatment concepts and ascertain which one exhibits superior biomechanical characteristics. Two different treatment concept models were constructed as described below: The initial model, LT4I, consisting of two mesial vertical implants positioned in lateral incisor regions and two distal tilted implants (45°) situated in second premolar regions of the maxilla. The second one, VS4I, includes two mesial vertical implants in lateral incisor regions and two vertically positioned short implants in second premolar regions. The proposed conjectures are as follows: (1) The level of stress in the peri-implant regions of short vertical implants is anticipated to be lower than that of tilted long ones in the maxilla. (2) Different occlusal force settings may lead to diverse biomechanical behavior on bone-implant interface.

## Materials and methods

### Model design

An examination using cone beam computerized tomography (CBCT, KaVo 3D eXam, Imaging Science International, USA, resolution 0.400 mm, field of view 130 mm, acceleration voltage 120 kV, beam current 5 mA, exposure time 8.9 s) was conducted to acquire the atrophic maxillary bony structure. In the posterior region of maxilla, the distance between the wall of maxillary sinus floor and the residual alveolar ridge was about 7.50 mm [18]. The CBCT data files, formatted as DICOM, were imported into Mimics 17 (Materialise, Leuven, Belgium) to reconstruct the maxillary model. The maxillary and overdenture models were then modified based on literature with professional software Geomagic Studio 12 (Geomagic Company, NC, USA). The finite maxillary model possessed dimensions of 15 mm in height, 50 mm in length and 90 mm in width. The thickness of cortical bone was 1 mm, and the residual bone was trabecular bone [19, 20]. The final prosthetic model ranges from the left first molar to the right first molar, including 12 teeth. Its dimension was 8 mm in height, 34 mm in length and 55 mm width.

**Fig. 1:**
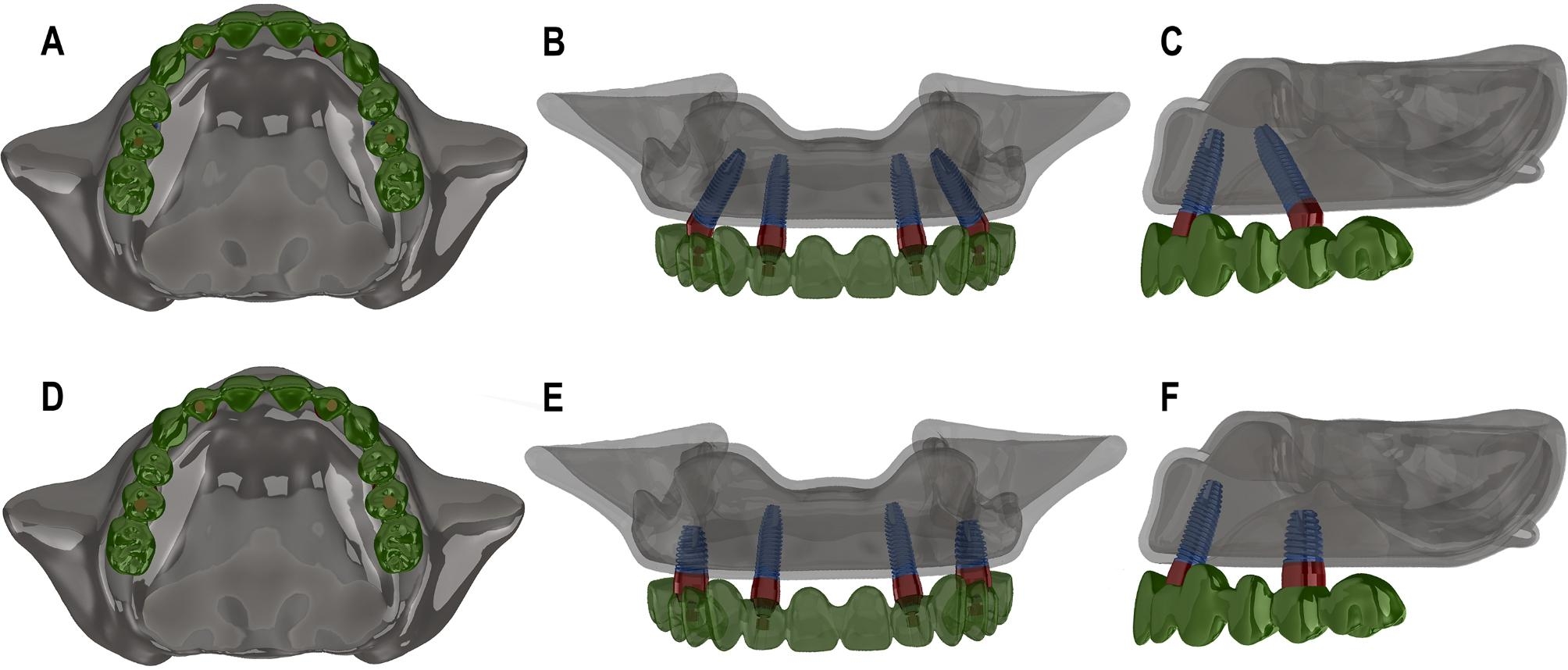
The prostheses and implant positioning on 3D models of the TL4I group (**A**: occlusal view; **B**: frontal view; **C**: lateral view) and VS4I group (**D**: occlusal view; **E**: frontal view; **F**: lateral view).

Nobel implants (Nobel Speed Groovy, Nobel Biocare, Yorba Linda, CA, USA), abutments and screws were chosen for this biomechanical analysis. The Nobel Biocare implants are recommended by Maló who first introduced the all-on-4 concept in 2003 [8]. The 3D geometry of the implants, abutments and screws were modeled with Solidworks 2014 (SolidWorks Corporation, Ve lizy-Villacoublay, France). The implants, abutments and prostheses were reconstructed jointly in the maxillary model according to the clinical situation. Two treatment concepts models were constructed, each involving four implants to support fixed prostheses: (1) TL4I group - two mesial implants (4.1 mm in diameter and 11.5 mm in length) were located vertically in lateral incisor regions and two distal implants (4.1 mm in diameter and 13.0 mm in length ) were located in second premolar regions and tilted at a 45-degree angle toward the anterior regions. (2) VS4I group - two mesial implants were the same as the ones of TL4I group and two distal short implants (5.0 mm in diameter and 7.0 mm in length) were placed vertically in second premolar regions. The prostheses utilized in both groups were the same. The locations and characteristic of the implants and abutments are showed in Table 1 and Fig. 1.

**Table 1:** The locations and characteristic of the implants and abutments

Groups	Implant location	Implants positioning	Implants features	Abutment features
TL4I	2-lateral Incisor	Vertical	Nobel Speed Groovy 11.5 × 4.1	Straight profile 4.1 × 4.0 mm
	2-2nd premolar	Inclined 45° to the distal	Nobel Speed Groovy 13.0 × 4.1	30° Angled profile 4.1 × 4.0 mm
VS4I	2-lateral Incisor	Vertical	Nobel Speed Groovy 11.5 × 4.1	Straight profile 4.1 × 4.0 mm
	2-2nd premolar	Vertical	Nobel Speed Groovy 7.0 × 5.0	Straight profile 5.0 × 4.0 mm

### Meshing procedure and material properties

**Fig. 2:**
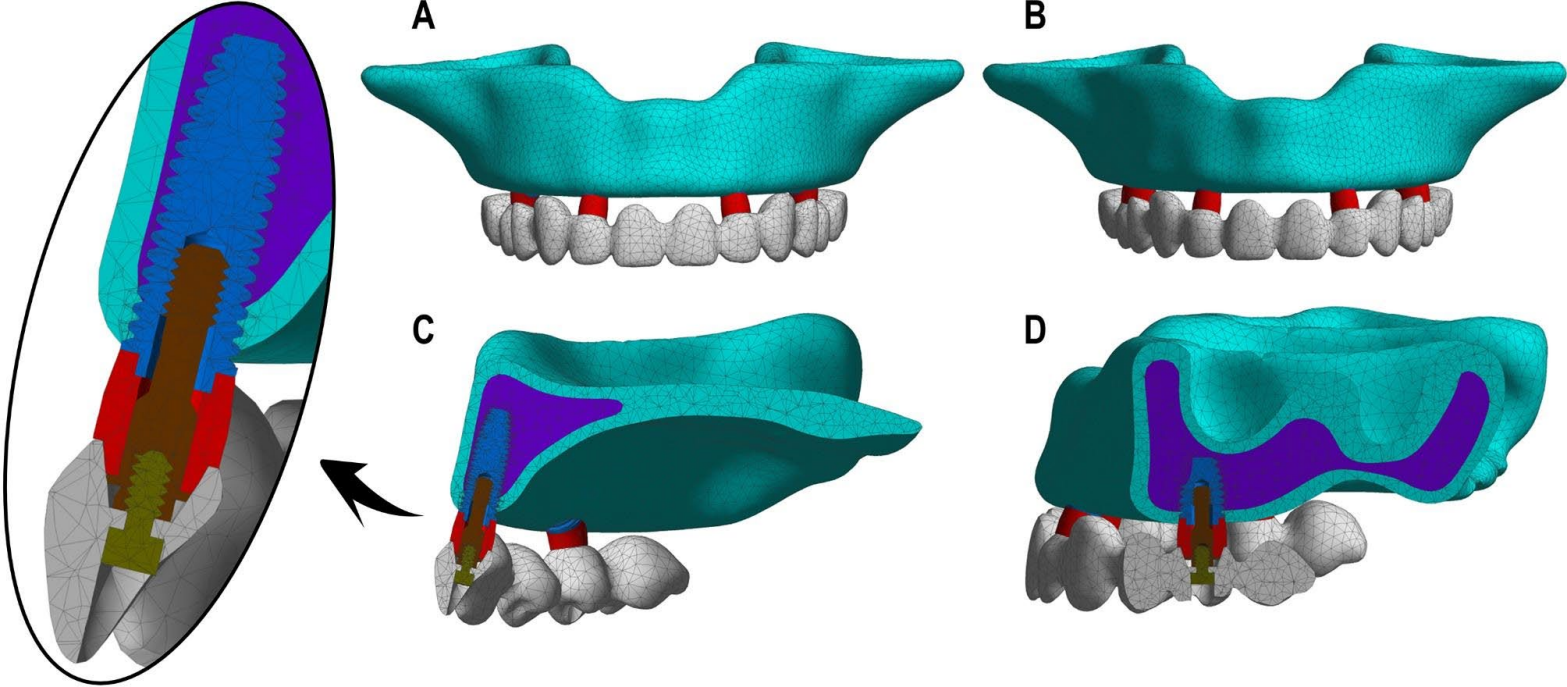
Finite element mesh for the TL4I group (**A**: frontal view; **C**: lateral view) and the VS4I group (**B**: frontal view; **D**: lateral view). The models consist of cortical bone, trabecular bone, implants, abutments, central screws, prosthesis screws, and prostheses.

The 3D models were imported into the ANSYS Workbench 17.0 software (Ansys Inc., Canonsburg, PA, USA) for generating meshes and defining material properties. The models were meshed using 10 node tetrahedral elements with a size of 1.5 mm. Furthermore, the tetrahedral elements were adjusted to accommodate all the small feature such as bone-implant interface. The TL4I groups presented a total of 156,854 elements and 274,862 nodes and the VS4I groups presented a total of 134,913 elements and 234,913 nodes. The mesh generation of groups are showed in Fig. 2. The models consist of cortical bone, trabecular bone, implants, abutments, central screws, prosthesis screws, and prostheses. Cortical and trabecular bone are anisotropic tissues [21]. The material properties of the rest models were assumed to be isotropic, homogeneous and linearly elastic and all material properties are showed in Table 2 [22].

**Table 2:** Material properties of models

Models	Material	Young’s modulus E (MPa)	Poisson’s ration	Shear modulus G (MPa)
Cortical bone	Cortical bone [19, 37]	Ex = 12,600 Ey = 12,600 Ez = 19,400	νxy = 0.300 νyz = 0.253 νxz = 0.253	Gxy = 4850 Gyz = 5700 Gxz = 5700
Trabecular bone	Trabecular bone [19, 37]	Ex = 1148 Ey = 210 Ez = 1148	νxy = 0.055 νyz = 0.010 νxz = 0.322	Gxy = 68 Gyz = 68 Gxz = 434
Implant Abutment Screws	Titanium alloy [38]	110,000	0.350	
Prosthesis	Zirconia [39]	210,000	0.310	

The vector of x mean the buccolingual direction, The vector of y mean the infero-superior direction and the vector of z mean the mesiodistal direction

### Boundary and loading conditions

The maxillary models were submitted to a strict fixation restriction in its upper area [1]. The implants were considered entirely osseointegrated [21]. And the cortical bone was bonded to the trabecular bone. The abutments were fixed in the implants through central screws. Their interfaces considered fixed together. The interfaces between prosthesis and abutments were also considered fixed together.

**Fig. 3:**
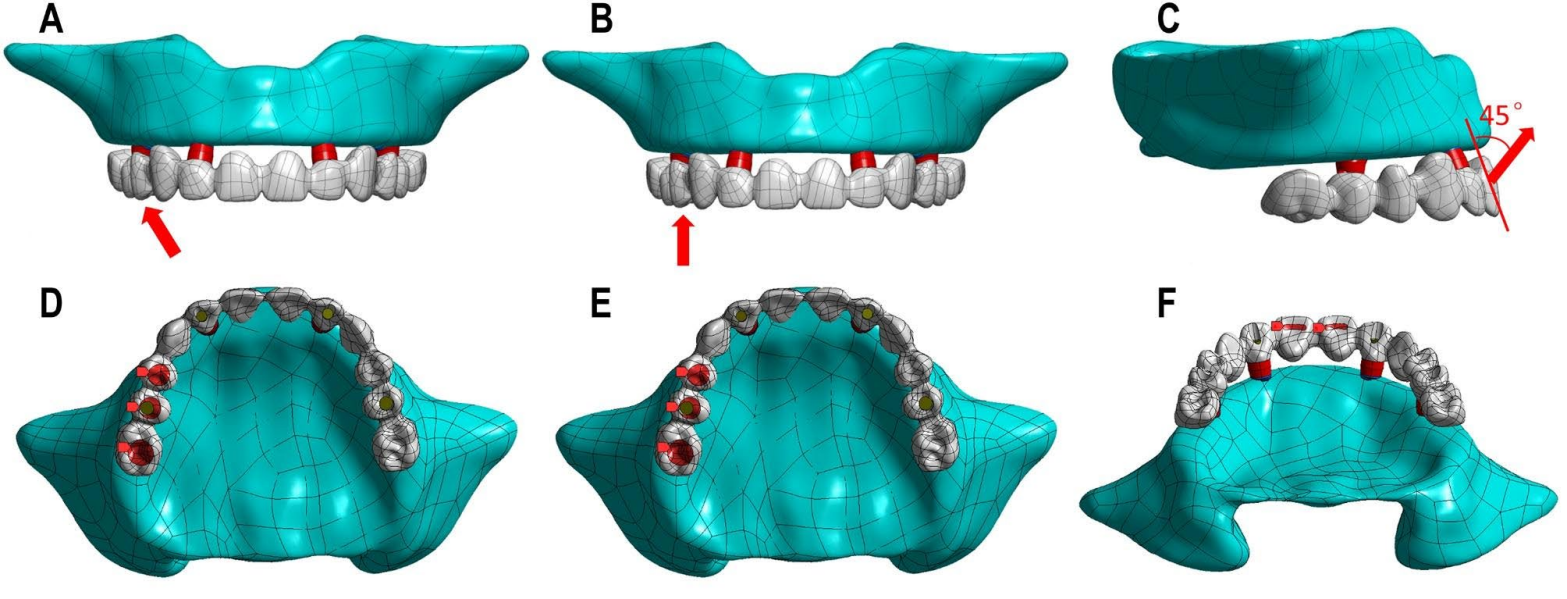
Three types of loading setting: (1) oblique loading on molars (**A**: loading direction; **D**: corresponding loading region); (2) vertical loading on molars (**B**: loading direction; **E**: corresponding loading region); (3) oblique loading on incisors (**C**: loading direction; **F**: corresponding loading region).

To simulate the occlusal force on the maxilla, three loading types were set in ANSYS Workbench 17.0. The first loading type was that tilting load of 150 N was imposed unilaterally on the posterior teeth with 30° in the buccal direction [23]. The teeth contacting with the loading surface were the right first premolar, right second premolar and right first molar. The second loading type was that vertical load of 150 N was imposed on the posterior teeth. The teeth contacting with the loading surface were the same to the first type [23]. The third loading type was that oblique load of 150 N was imposed on bilateral central incisors, at a 45° angle with the long axis of the incisors [24]. The loading types are presented in Fig. 3.

### Stress analysis

FEA was analyzed by ANSYS Workbench 17.0. To distinguish the tensile stress from the compressive stress, the maximum and minimum principal stresses (σ_max_ and σ_min_) were selected as the stress output of the cortical and trabecular bone [5]. The mesial, distal, buccal, and lingual σ_max_ and σ_min_ values of the bone around the implant were recorded and analyzed [25]. Von Mises stress(σ_VM_) was selected for implants.

## Result

### Oblique load on molars

**Fig. 4:**
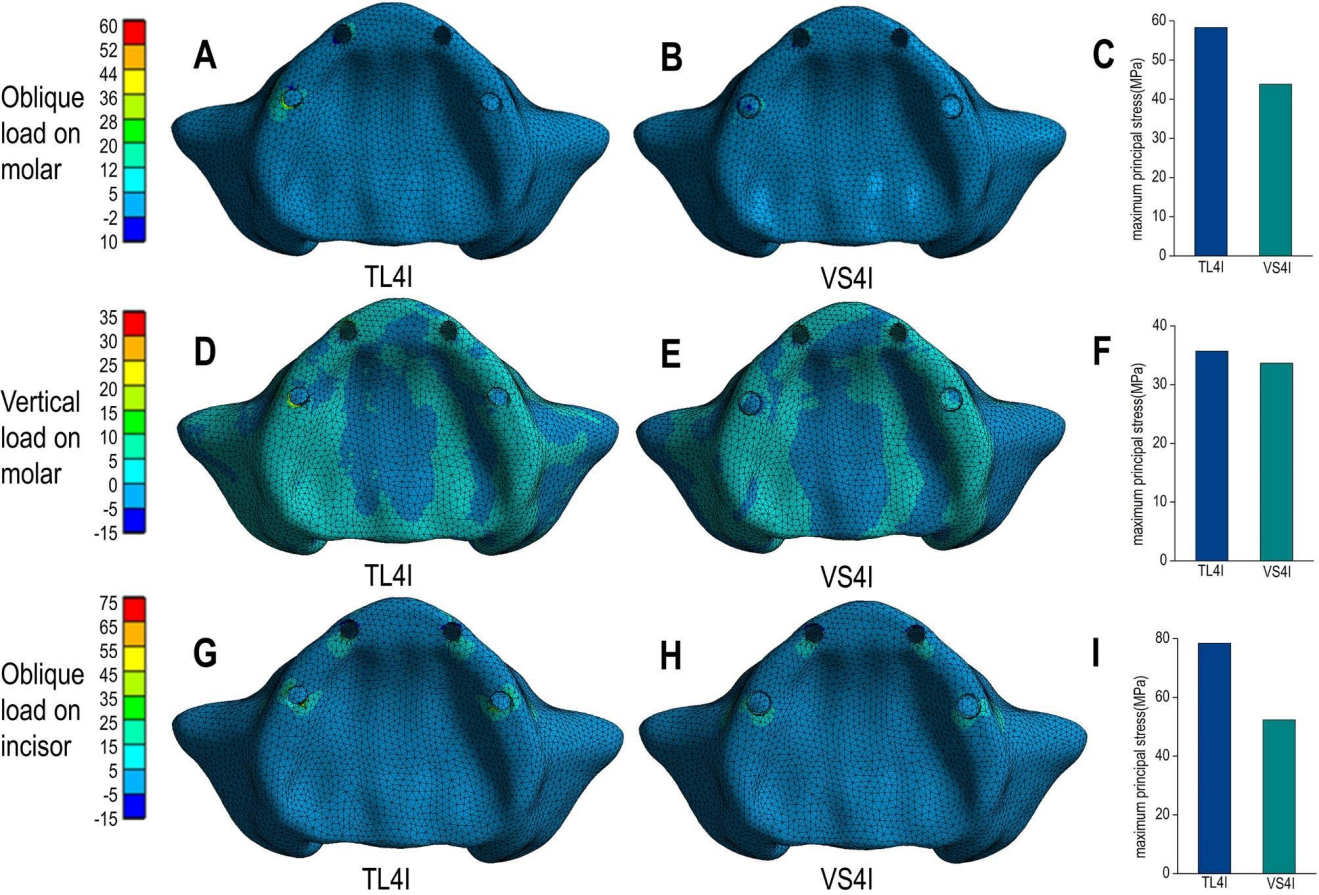
Maximum principal stress (σ_max_) distribution (MPa) in the cortical bone for the TL4I group (**A**: oblique load on molars; **D**: vertical load on molars; **G**: oblique load on incisors) and VS4I group (**B**: oblique load on molars; **E**: vertical load on molars; **H**: oblique load on incisors). The peak σ_max_ values of three loading types (**C**: oblique load on molars; **F**: vertical load on molars; **I**: oblique load on incisors) for the two groups.

**Fig. 5:**
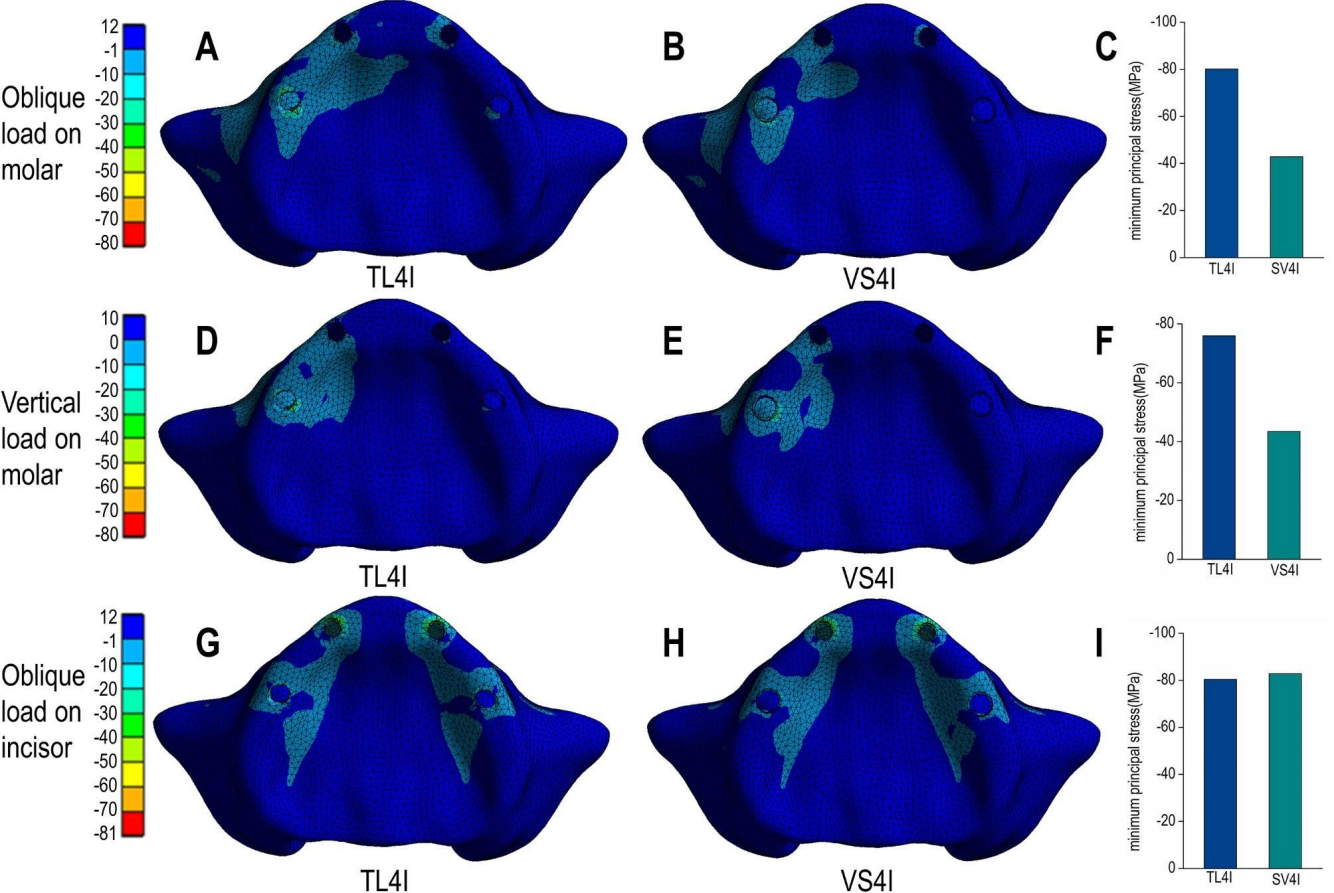
Minimum principal stress (σ_min_) distribution (MPa) in the cortical bone for TL4I group (**A**: oblique load on molars; **D**: vertical load on molars; **G**: oblique load on incisors) and VS4I group (**B**: oblique load on molars; **E**: vertical load on molars; **H**: oblique load on incisors). The peak σ_min_ values of three loading types (**C**: oblique load on molars; **F**: vertical load on molars; **I**: oblique load on incisors) for the two groups.

**Fig. 6:**
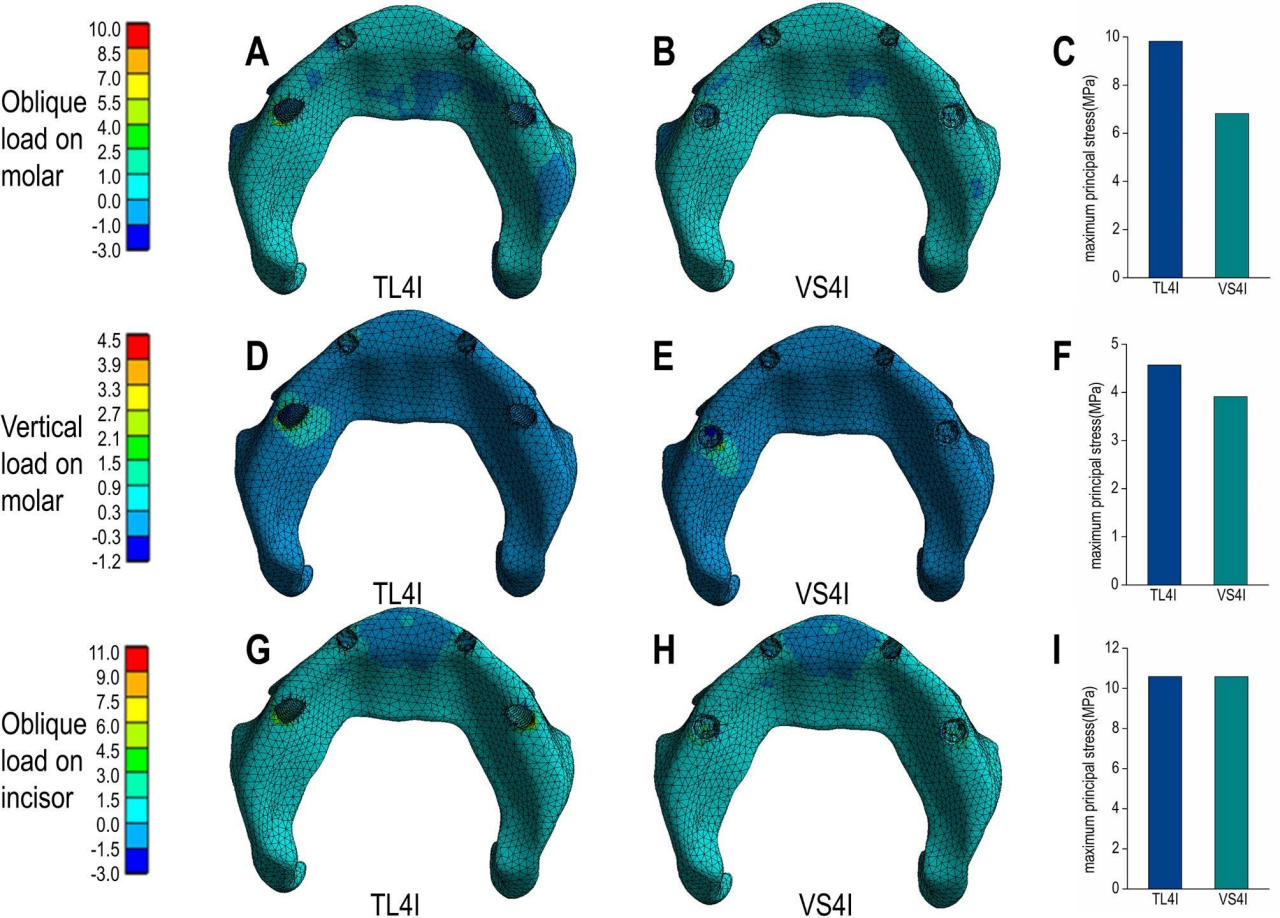
Maximum principal stress (σ_max_) distribution (MPa) in the trabecular bone for TL4I group (**A**: oblique load on molars; **D**: vertical load on molars; **G**: oblique load on incisors) and VS4I (**B**: oblique load on molars; **E**: vertical load on molars; **H**: oblique load on incisors) groups. The peak σ_max_ values of three loading types (**C**: oblique load on molars; **F**: vertical load on molars; **I**: oblique load on incisors) for the two groups.

**Fig. 7:**
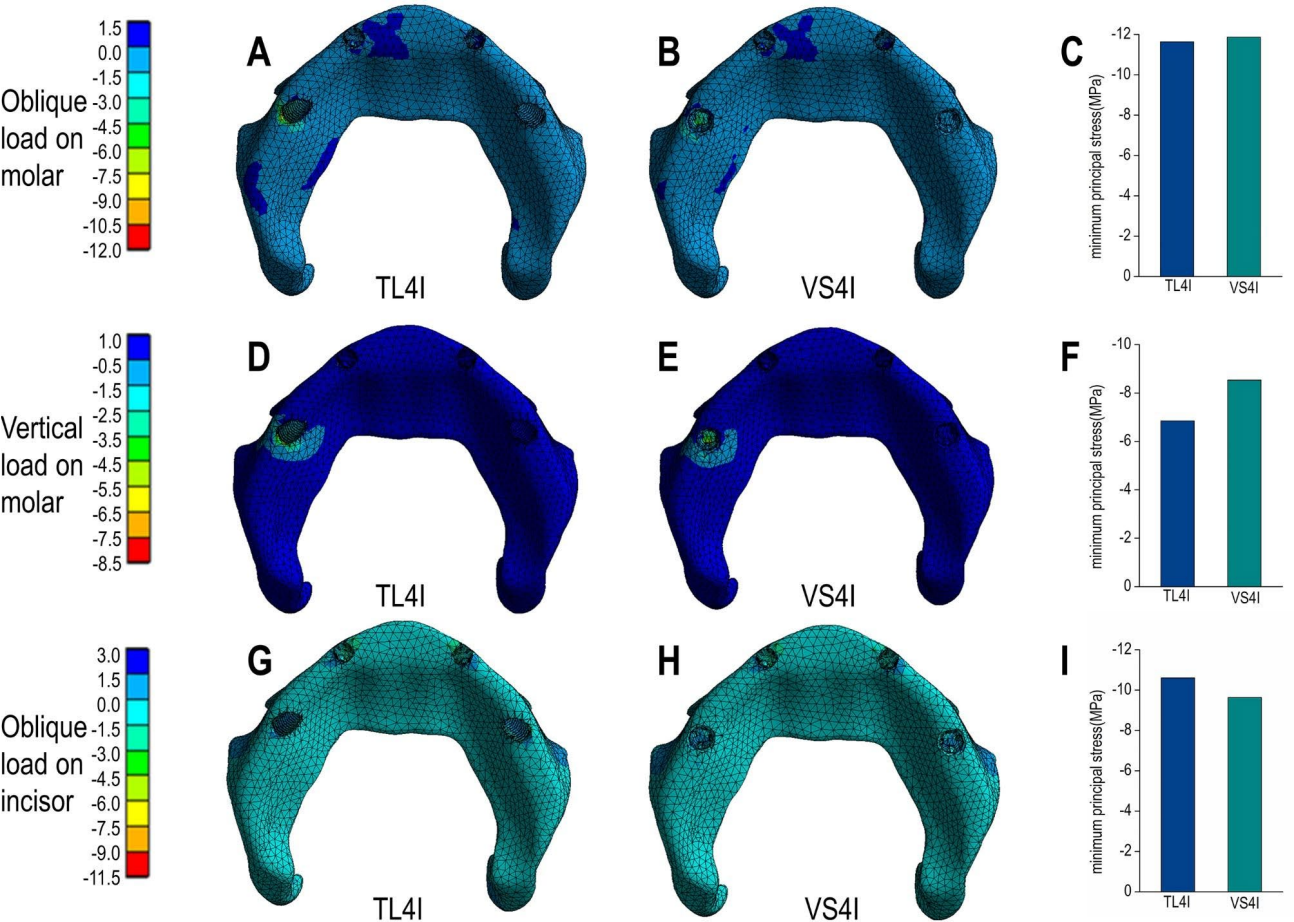
Minimum principal stress (σ_min_) distribution (MPa) in the trabecular bone for TL4I group (**A**: oblique load on molars; **D**: vertical load on molars; **G**: oblique load on incisors) and VS4I group (**B**: oblique load on molars; **E**: vertical load on molars; **H**: oblique load on incisors). The peak σ_min_ values of three loading types (**C**: oblique load on molars; **F**: vertical load on molars; **I**: oblique load on incisors) for the two groups.

**Fig. 8:**
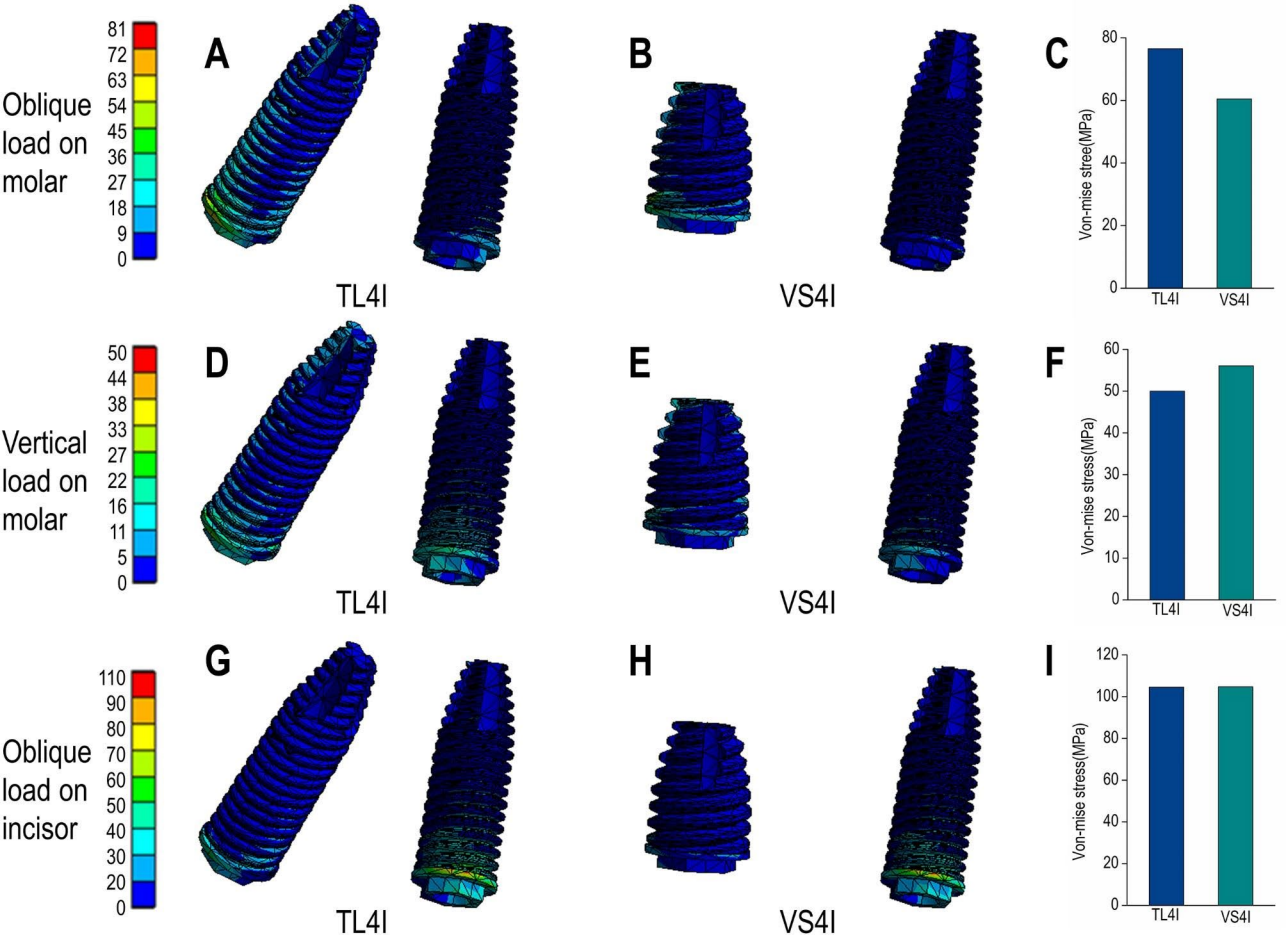
Von Mise stress (σ_VM_) distribution (MPa) in the trabecular bone for TL4I group (**A**: oblique load on molars; **D**: vertical load on molars; **G**: oblique load on incisors) and VS4I group (**B**: oblique load on molars; **E**: vertical load on molars; **H**: oblique load on incisors). The peak σ_VM_ values of three loading types (**C**: oblique load on molars; **F**: vertical load on molars; **I**: oblique load on incisors) for the two groups.

Under oblique loading on molars, TL4I group exhibited higher stress concentration than VS4I groups. The σ_max_ and σ_min_ values of bone are showed in Table 3. Both groups demonstrated a similar distribution of stress, with the σ_max_ and σ_min_ mainly concentrated on the cortical bone. Particularly, the peak σ_max_ and σ_min_ value were observed around the distal implant, especially in the distal and buccal areas. In addition, the TL4I group exhibited a peak σ_max_ value of 58.33 MPa in the cortical bone, approximately 1.33 times higher than that of the VS4I group. Similarly, the peak σ_min_ value of the cortical bone was 80.16 MPa in TL4I group, approximately 1.87 times than that in VS4I group (Figs. 4A-C and 5A-C). The stress distribution in the trabecular bone was found to be similar in both groups (Figs. 6A-C and 7A-C). In terms of implants, the σ_VM_ mainly concentrated on the neck proportion. The maximum σ_VM_ value of implant in TL4I group was 76.49 MPa, which was 26.5% higher than that of the VS4I group (Fig. 8A-C).

**Table 3:** Summary of data obtained from TL4I and VS4I groups under oblique load on molars

Structure		Position	TL4I			VS4I		
			Peak value	Right second premolar region	Right lateral incisors region	Peak value	Right second premolar region	Right lateral incisors region
Cortical bone	Maximum principal stress	Buccal	58.33	47.02	1.432	43.83	4.486	1.762
		Palatal		12.53	0.250		5.430	1.010
		Mesial		6.165	8.611		2.253	8.124
		Distal		46.77	3.680		23.08	1.466
	Minimum principal stress	Buccal	-80.16	-46.96	-1.773	-42.86	-21.18	-0.746
		Palatal		-46.93	-5.614		-3.026	-3.036
		Mesial		-2.962	-1.427		-2.991	-0.720
		Distal		-9.523	-13.11		-10.07	-7.625
Concellous bone	Maximum principal stress	Buccal	9.820	1.138	0.047	6.816	0.854	0.062
		Palatal		0.851	0.129		0.124	0.173
		Mesial		0.275	0.649		0.278	0.057
		Distal		8.829	0.016		0.270	0.323
	Minimum principal stress	Buccal	-11.64	-3.019	-0.083	-11.87	0.211	-0.051
		Palatal		-1.135	-0.114		-0.530	-0.028
		Mesial		-0.047	-0.131		-0.100	-0.020
		Distal		-6.148	-0.417		-1.800	-0.276
Implant	Von-Mise stress		76.49			60.47		

### Vertical load on molars

Under condition of vertical loading on molars, the TL4I group also showed a higher degree of stress concentration compared to the VS4I group. The σ_max_ and σ_min_ of bone are presented in Table 4. Similarly, the σ_max_ and σ_min_ were primarily concentrated in the cortical bone. The peak σ_max_ and σ_min_ were observed on distal implant-bone interface areas. In the cortical bone, the peak σ_max_ value of the TL4I group was 35.72 MPa, which was similar to that of VS4I group (Fig. 4D-F). However, the peak σ_min_ value of the TL4I group was 75.95 MPa, which was much higher compared to the VS4I group (Fig. 5D-F). As for the trabecular bone, similar stress levels were observed in both groups (Figs. 6D-F and 7D-F). In addition, the implants in the VS4I group exhibited slightly higher σ_VM_ values than that in the TL4I group (Fig. 8D-F).

**Table 4:** Summary of data obtained from TL4I and VS4I groups under vertical load on molars

Structure		Position	TL4I			VS4I		
			Peak value	Right second premolar region	Right lateral incisors region	Peak value	Right second premolar region	Right lateral incisors region
Cortical bone	Maximum principal stress	Buccal	35.72	17.97	8.856	33.66	0.997	6.203
		Palatal		2.195	-1.163		-6.549	-1.007
		Mesial		0.653	9.200		1.298	5.356
		Distal		23.18	-0.549		11.63	0.062
	Minimum principal stress	Buccal	-75.95	-19.60	0.046	-43.48	-3.993	-0.440
		Palatal		-66.03	-8.492		-28.75	-2.552
		Mesial		-8.538	0.934		-1.245	0.383
		Distal		-1.282	-11.14		-16.27	-5.025
Concellous bone	Maximum principal stress	Buccal	4.569	2.250	0.308	3.912	0.370	0.165
		Palatal		1.574	-0.028		3.467	0.055
		Mesial		-0.018	0.350		0.342	0.176
		Distal		3.968	-0.144		0.247	-0.117
	Minimum principal stress	Buccal	-6.846	-0.788	-0.119	-8.539	0.210	0.006
		Palatal		-1.540	-0.206		-0.828	-0.095
		Mesial		-0.021	-0.004		-0.022	-0.020
		Distal		-5.865	-0.377		-1.309	-0.125
Implant	Von mise stress		49.99			56.07		

### Oblique load on incisors

When deliberating upon the oblique load on incisors, notable distinctions emerged between the TL4I and VS4I groups. The σ_max_ and σ_min_ primarily localized within the cortical bone surrounding the distal implants in the TL4I group, whereas in the VS4I group, they concentrated at the interface area between the mesial implant and bone. The σ_max_ and σ_min_ values of bone are showed in Table 5. In the cortical bone, the TL4I group exhibited a peak σ_max_ value of 78.42 MPa, surpassing that of the VS4I group (Fig. 4G-I). The peak σ_min_ value of TL4I groups was 80.36 MPa, closely resembling the VS4I group (Fig. 5G-I). Furthermore, comparable stress levels were observed in the trabecular bone (Figs. 6G-I and 7G-I). The σ_VM_ of the implants exhibited similarity between the two groups, with values of 104.51 MPa and 104.76 MPa, respectively (Fig. 8G-I).

**Table 5:** Summary of data obtained from TL4I and VS4I groups under vertical load on incisors

Structure		Position	TL4I			VS4I		
			Peak value	Right second premolar region	Right lateral incisors region	Peak value	Right second premolar region	Right lateral incisors region
Cortical bone	Maximum principal stress	Buccal	78.42	29.14	-5.718	52.33	5.207	-2.681
		Palatal		78.42	4.743		7.001	6.593
		Mesial		-2.691	-4.679		0.482	-0.196
		Distal		6.084	19.01		38.88	18.93
	Minimum principal stress	Buccal	-80.36	-2.154	-33.24	-82.83	-10.87	-22.09
		Palatal		-0.877	-7.318		1.159	-11.32
		Mesial		-13.85	-54.46		-4.582	-67.60
		Distal		-8.788	3.931		-14.92	0.221
Concellous bone	Maximum principal stress	Buccal	10.59	1.311	-0.005	10.58	0.102	0.262
		Palatal		0.462	0.256		-0.048	0.140
		Mesial		-0.114	-0.173		-0.024	-0.004
		Distal		5.867	2.557		1.142	0.615
	Minimum principal stress	Buccal	-10.61	-2.114	-0.284	-9.638	-0.059	-0.269
		Palatal		-0.051	0.060		-0.237	-0.156
		Mesial		-0.513	-1.886		-0.535	-2.088
		Distal		0.757	0.030		-0.042	-0.056
Implant	Von-mise stress		104.5			104.7		

## Discussions

In this FEA study, the CBCT was taken to construct the implantation models in a moderate atrophic maxilla. The objective was to compare the biomechanical behavior of two different treatment concepts - a fixed prosthesis supported by four implants in an edentulous and moderate atrophic maxilla [5]. While the application of fewer implants to support the prosthesis may result in cost reduction, the decrease of bone volume poses challenges for implant placement [26]. At present, the “all-on-4” treatment concept has been recognized for its success and practicality in scenarios where limited bone volume is present due to bone resorption and sinus pneumatization in the posterior region of the maxilla [27]. Furthermore, the utilization of short implants further serves as a feasible alternative with advantageous clinical results. Thus, it is necessary to conduct a biomechanical evaluation of the two treatment concepts.

Fellippo Ramos Verri et al. demonstrated that the application of simplified models for the implant surface can alter the distribution of stress and strain on the cortical bone [28]. Furthermore, a slight simplification of the implants, specifically the threads, has no impact on the distribution of stress and strain on the cortical bone tissue [28]. Therefore, a slight simplification of the implants was used in this study. To accurately simulate the oral clinical situation, abutments, central screws and prosthesis screws were established in this study. Furthermore, the anisotropic nature of both the cortical and trabecular bone, which cannot be disregarded in numerical simulations, was taken into consideration [14]. Moreover, various stress and strain measurements, such as von-Mises stress, maximum and minimum principal stress, and equivalent elastic strain, are commonly utilized for calculating and evaluating the biomechanical behaviors of both bones and implants. The maximum principal stress is usually used to observe tensile stress, while the minimum principal stress is used for compressive stress. This approach is fitting for examining the biomechanical behavior of bones due to their ductility and brittleness [5]. Titanium, as a ductile material, the von-Mises stress was selected for analysis. In addition, a convergence analysis is a crucial step in validating the reliability and accuracy of FEA results. However, convergence analysis may not be necessary for FEA, particularly when the following conditions are met: (1) Sufficiently refined mesh has been used to capture the key physical phenomena; (2) The obtained results are acceptable within the scientific accuracy and are consistent with existing analytical results. Therefore, convergence analysis was not performed in our study.

In all groups, the stress values of the cortical bone surpassed those of the trabecular bone. This phenomenon can be explained by the fact that cortical bones have a higher elastic modulus compared to trabecular bone. Thus, cortical bones exhibit greater strength and resilience against deformation. Consequently, cortical bones are subjected to elevated loads in clinical scenarios, distinguishing they from trabecular bones [29, 30]. Within this study, three types of loads were considered to simulate the oral mastication. Similar to previous studies, the oblique load models demonstrated higher levels of stress in comparison to the vertical load models [25, 31]. Furthermore, upon considering the oblique load on incisors, contrasting stress distributions were observed between the two groups. In the TL4I group, σ_max_ and σ_min_ were mainly concentrated on cortical bone around the distal implants, owing to the implant tilting. This result is in accordance with previous studies [5, 32].

In the realm of three different loading scenario, higher stresses were observed in the TL4I group compared to the VS4I group [30, 32]. When subjected to the oblique loading on molars, the TL4I group demonstrated a peak σ_max_ value of 58.33 MPa, approximately 1.33 times greater than that of the VS4I group. Under vertical loading on molars, the TL4I group displayed a peak σ_min_ value of 75.96 MPa, surpassing that of the VS4I group (43.48 MPa). Similarly, when subjected to an oblique load on incisors, the TL4I group exhibited a peak σ_max_ value of 78.42 MPa, surpassing that of the VS4I group (52.33 MPa). This phenomenon can be attributed to the larger diameter of the short implants and the oblique of the long implants [16, 30]. Moreover, the uppermost portion of the implants, roughly 2-3 mm in length, plays a significant role in transferring the primary load to the surrounding bone tissue, aligning with the findings of other relevant studies [14, 15]. These findings may serve as a basis for selecting short implants assuming they are stably anchored in the bone. However, it is necessary to emphasize that the predictability of short implants is contingent upon various factors, such as implant design, placement protocol, remaining bone height and volume, occlusion conditions, and patient oral hygiene [1, 33].

Notably, surpassing the constraints of physiological limits (ultimate bone strength), when σ_min_ reaches over 170 MPa or σ_max_ exceeds 100 MPa, overloading of cortical bone may occur [1, 34]. In this study, the observed values in both treatment concepts were lower compared to those associated with histopathological bone. It can be concluded that both concepts are viable. These findings provide an explanation for the elevated success rate of the all-on-4 treatment method. It is imperative to ensure that the σ_VM_ value of the implants remains below 550 MPa, which corresponds to the yield strength of titanium implants. Failure to adhere to this threshold may result in implantation failure [35, 36]. Notably, none of the implants surpassing the σ_VM_ threshold of 550 MPa in this research.

There are still some limitations in this study. While the inclusion of static loads has been taken into account to represent occlusal loads, the existence of chewing movement necessitates the implementation of dynamic load simulations in future studies [5]. Moreover, it is noteworthy that the TL4I (all-on-4) treatment concept is typically applied to immediate loading rather than delayed loading in a clinical context [10]. However, in this study, the implants are fully osseointegrated, which means that the obtained results are more suitable for delayed loading. In conclusion, these findings provide us some clinical guidance, further longitudinal follow-up and randomized clinical trials are necessary to confirm the predictability of short implants.

## Conclusion

Based on the findings of this study, both treatment concepts can be applied in edentulous and moderate atrophic maxilla. Compared to tilted implants, short implants can transmit less occlusal force to the supporting tissues. By optimizing the implant design and implantation procedure, short implants may be crucial to the restoration and rehabilitation of atrophic maxilla.

## Data Availability

The data presented in this study are available on request from the corresponding author.
